# Quantum Beats between Spin-Singlet and Spin-Triplet Interlayer Exciton Transitions in WSe_2_–MoSe_2_ Heterobilayers

**DOI:** 10.1021/acs.nanolett.4c00831

**Published:** 2024-04-19

**Authors:** Mehmet Atıf Durmuş, Ibrahim Sarpkaya

**Affiliations:** † Bilkent University UNAM − National Nanotechnology Research Center, Ankara 06800, Turkey

**Keywords:** quantum beats, coherence, interlayer exciton, heterobilayer, singlet, triplet, dephasing time

## Abstract

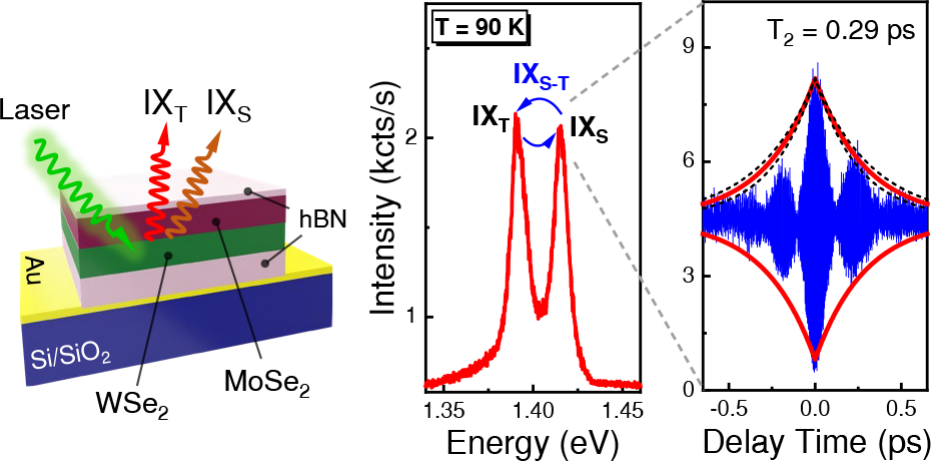

The long-lived interlayer excitons (IXs) of semiconducting transition metal dichalcogenide heterobilayers are prime candidates for developing various optoelectronic and valleytronic devices. Their photophysical properties, including fine structure, have been the focus of recent studies, and the presence of two spin states, namely, spin-singlet and spin-triplet, has been experimentally confirmed. However, the existence of the interaction between these states and their nature remains unknown to date. Here, we demonstrate the presence of coherent coupling between the spin-singlet and spin-triplet IXs of a WSe_2_–MoSe_2_ heterobilayer utilizing quantum beat spectroscopy via a home-built Michelson interferometer. As a clear signature of coherent coupling, the quantum beat signal has been observed for the first time between closely spaced transitions of IXs. The observed strong damping of the quantum beat signals with fast dephasing times of 270–400 fs indicates that fluctuations giving rise to inhomogeneous broadening in the photoluminescence emission of these states are uncorrelated.

Van der Waals heterostructures of two-dimensional semiconducting transition metal dichalcogenides (TMDCs) have been extensively explored in recent years to develop next-generation efficient optoelectronic,^1,2^ quantum photonic,^3^ and valleytronic devices.^4^ Due to type-II band alignment and ultrafast charge transfer between the individual monolayers of TMDC heterobilayers, spatially separated Coulomb-bound interlayer excitons (i.e., an electron in one layer and a hole in the other) can be formed in addition to intralayer excitons of individual monolayers.^5,6^ The photophysical properties of these interlayer excitons (IXs), such as their spontaneous emission lifetimes,^6−8^ temporal coherence,^9,10^ and diffusion dynamics,^11^ have been studied in detail using various experimental techniques. The fine structure of IXs in TMDC heterobilayers has also been investigated, and the presence of two different IX states, namely, the spin-singlet (spin-conserved) and spin-triplet (spin-forbidden), has been theoretically predicted.^12^ The existence of those states in WSe_2_–MoSe_2_ heterobilayers was demonstrated in recent experimental studies^9,13−16^ via corresponding photoluminescence (PL) emission signals. The experimentally observed near-unity valley polarization,^14,17,18^ long spontaneous emission lifetimes,^18^ and optical^19^ and electrical^4,15,18^ tunability for the PL emission of these IX states make them promising candidates for future valleytronic applications. The opposite polarization behavior observed in the PL emission of singlet and triplet IXs^4,14,18^ could also be utilized for the development of valley polarization switches in exciton-based devices. However, for their efficient use in the proposed applications of valleytronics, one needs to also know whether there is an interaction or correlation between these states. The presence of this interaction and its nature, if there is, remains unknown. Quantum beat spectroscopy^20^ can be utilized to answer these open questions. Quantum beats as a periodic oscillation in the time domain can be observed due to the excitation of coherent superposition of two energetically closed transitions. It has been utilized for the closely spaced different transitions of various material platforms, such as quantum dots,^21^ quantum wells,^22−26^ monolayers of 2D TMDCs,^27−29^ and ReS_2_.^30^ The coherent interaction between the intralayer excitons of MoSe_2_ and WSe_2_ in a WSe_2_–MoSe_2_ heterobilayer has also been shown in a recent study.^31^ However, it has yet to be performed for the different spin states of the interlayer excitons in the TMDC heterobilayers.

Here, we studied the coherent coupling between the spin-singlet and spin-triplet interlayer excitons of a WSe_2_–MoSe_2_ heterobilayer utilizing quantum beat spectroscopy via a home-built free-space Michelson interferometer. As a clear signature of coherent coupling, the quantum beating signal has been observed for the first time between the two spin states of the IXs in the time domain. The quantum beat dephasing times up to 400 fs were measured. The calculated energy difference obtained by the measured beating period of 195 ± 17 fs is in close agreement with the energy separation between the PL emission peaks of spin-singlet and spin-triplet interlayer excitons in the time-integrated PL spectrum and further confirms the coherent nature of the coupling between these states.

To investigate the coherence properties of both spin-singlet (IX_S_) and spin-triplet (IX_T_) interlayer excitons and the nature of the interaction between them, we have fabricated hexagonal boron nitride (hBN) encapsulated WSe_2_–MoSe_2_ heterobilayers with a nearly 60° twist angle using the combination of well-known viscoelastic dry transfer technique^32^ and edge identification method.^33^ The details of the fabrication process of the heterobilayer can be found in the materials and methods section of the Supporting Information, and in our previous work.^9^ The optical microscope image of a typical hBN-encapsulated WSe_2_–MoSe_2_ heterobilayer on top of an Au-coated (∼100 nm thick) substrate is shown in Figure 1a. In this heterobilayer, the 60° twist angle (AB stacking) allows the alignment of the K valley of the WSe_2_ monolayer (i.e., valence band maximum) with the K′ valley of MoSe_2_ (i.e., conduction band minimum) in momentum space. In contrast to the TMDC monolayer case,^34,35^ breaking of the out-of-plane mirror symmetry in a WSe_2_–MoSe_2_ heterobilayer allows for the observation of not only the spin-singlet but also the spin-triplet IX PL emission.^12^ The schematic of the type-II band alignment of the WSe_2_–MoSe_2_ heterobilayer and the formation basics of the spin-singlet and spin-triplet IXs are shown in Figure 1b. As we further discuss in the following sections, the PL intensities of the IX_S_ and IX_T_ peaks must be close to each other to resolve the quantum beats efficiently in a PL interferogram. Therefore, we performed our experiments at 90 K, where the intensities of the IX_T_ and IX_S_ emissions were nearly equal. The PL spectrum of the studied heterobilayer at 90 K is shown in Figure 1c. The complete evolution of the PL at different temperatures is given in Supporting Information, Figure S1. In addition to strongly suppressed intralayer exciton emissions of MoSe_2_ and WSe_2_ monolayers in the 1.6 and 1.7 eV regions, two low-energy pronounced PL emission peaks have been observed around 1.391 and 1.415 eV, which belong to IX_T_ and IX_S_, respectively. The measured energy separation of ∼24 meV between the IX_S_ and IX_T_ peaks corresponds to spin–orbit coupling-induced conduction band splitting in MoSe_2_^36^ and is similar to what has been observed in previous studies.^4,13,14,37^

**Figure 1:**
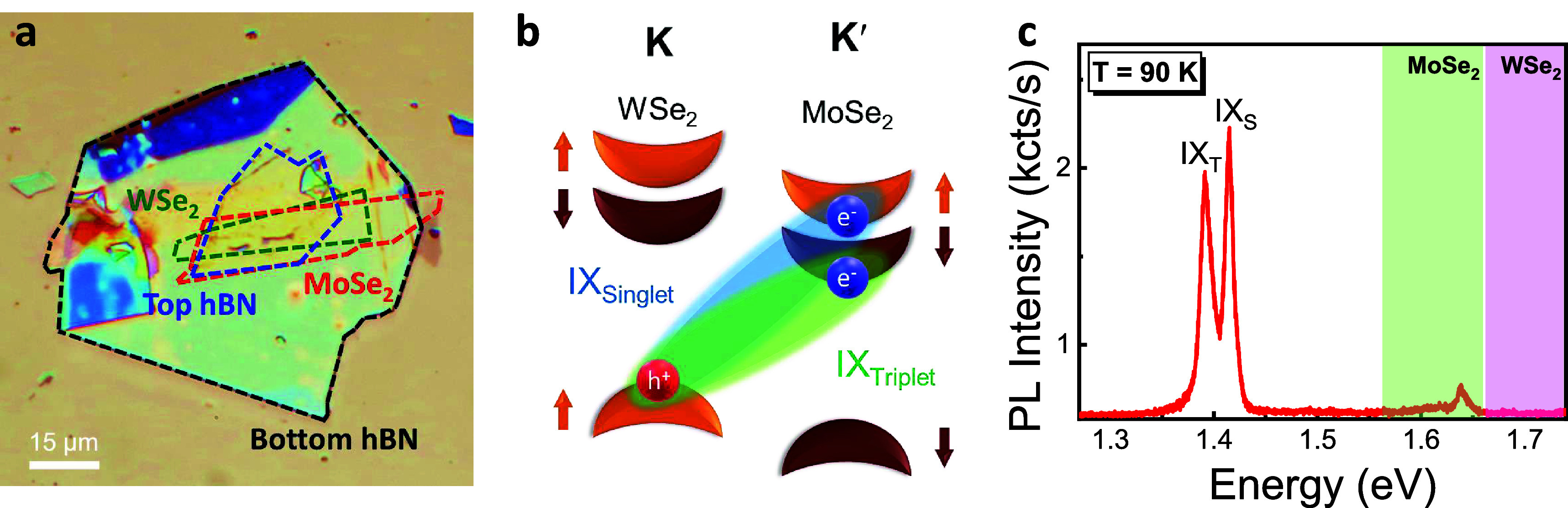
Interlayer exciton and PL characteristics of the WSe_2_–MoSe_2_ heterobilayer. (a) Optical microscope image of the hBN encapsulated WSe_2_–MoSe_2_ heterobilayer. (b) Schematic of type-II band alignment of the WSe_2_–MoSe_2_ heterobilayer, showing the spin-singlet and spin-triplet interlayer excitons. The blue and green areas represent the formation of IX_S_ and IX_T_, whereas orange and brown colored arrows represent spin-up and spin-down configurations, respectively. (c) Low-temperature photoluminescence (PL) spectrum of the WSe_2_–MoSe_2_ heterobilayer with the IX_T_ and IX_S_ emissions appearing at 1.391 and 1.415 eV, respectively, under 532 nm CW laser excitation with an incident power of *P* = 400 μW. Green and purple boxes show the region of intralayer exciton PL emissions of MoSe_2_ and WSe_2_ monolayers, respectively.

To confirm the assignments of IX_T_ and IX_S_ emissions, we have further performed magneto-PL spectroscopy and extracted the corresponding Landé *g*-factors at 90 K. We used a linearly polarized 532 nm continuous wave (CW) laser as an excitation source and collected PL emissions as a function of applied magnetic field perpendicular to the heterobilayer (i.e., in Faraday geometry). As shown in Figure 2a, both IX PL peaks split into two as the applied magnetic field strength increases due to the Zeeman shift of the corners (K and K′ valleys) of the hexagonal Brillouin zone of WSe_2_–MoSe_2_ heterobilayer. The Zeeman splitting can be defined as |Δ*E*_IX-S,T_| = *g*μ_B_*B*, where *g* is the effective Landé *g*-factor, μ_B_ is the Bohr magneton (∼58 μeV/T), and *B* is the strength of the applied magnetic field. With this expression, the calculated Valley-Zeeman splitting over applied magnetic field points can be linearly fitted, which gives *g*-factor values of 12.39 ± 0.37 for IX_S_ and 15.42 ± 0.49 for IX_T_. These experimentally measured *g*-factor values of IX_S_ and IX_T_ from our WSe_2_–MoSe_2_ heterobilayer are in excellent agreement with the results of previous experimental and theoretical studies.^13,37−40^ It also confirms the nearly 60° stacking angle of our heterobilayer.

**Figure 2:**
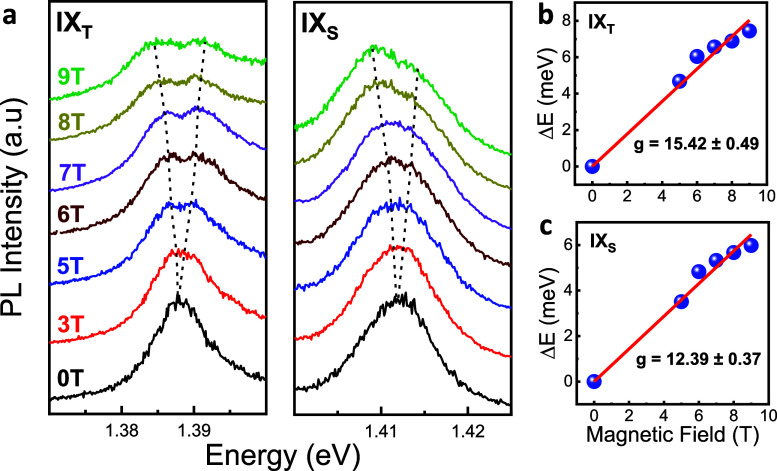
Valley-Zeeman splitting of the spin-triplet and spin-singlet IX emission in the WSe_2_–MoSe_2_ heterobilayer. (a) The PL spectra of IX_T_ and IX_S_ as a function of the strength of an applied out-of-plane magnetic field under 532 nm CW laser excitation. The Valley-Zeeman splitting measured for IX_T_ (b) and IX_S_ (c) with effective *g*-factors of 15.42 ± 0.49 and 12.39 ± 0.37, respectively.

Having confirmed the assignments of IX_T_ and IX_S_, we next study the coherence properties of both IX_T_ and IX_S_ in the time domain. To measure the decay of coherence of IX_T_ and IX_S_ in the time domain (*T*_2_) separately and probe whether there is a coherent coupling between them (i.e., the signature of quantum beating), we recorded the first-order autocorrelation function *g*^(1)^(τ) with a home-built Michelson interferometer. The free-space Michelson interferometer consists of a 50:50 beam splitter and two movable retroreflectors (one fixed on the DC motor stage, the other one on the piezo stage) to create a delay in time. More details about the interferometer setup can be found in our previous study.^9^ We first investigate the dephasing properties of IX_T_ and IX_S_ emissions individually by allowing emission from only one of them to the interferometer. Using 10 nm bandpass filters, we spectrally filter these emissions before they enter the interferometer. Figure 3a,b shows the spectrally filtered PL spectra of IX_S_ and IX_T_, respectively, at 90 K. Figure 3c,d shows the corresponding PL interferogram of the first-order coherence function *g*^(1)^(τ) of IX_T_ and IX_S_. At near-zero delay time, the fringe contrast with the value of ∼90% becomes pronounced, obtained with the retroreflector fixed on the piezo-stage with a stepping resolution of 20 nm. The visibilities of the fringes at various delay times are calculated by using their fringe maxima (*I*_max_) and minima (*I*_min_) in the envelope function of the interferogram in the visibility expression, . The visibility decay as a function of delay time can be fitted to determine the dephasing time (*T*_2_) of IX_T_ and IX_S_ emissions. Figure 3e,f shows the resulting visibility decay of IX_T_ and IX_S_ in a semilog plot. The initial kicks in both figures in the first ∼150 fs part indicate that both PL emission spectra are inhomogeneously broadened and deviate from the monoexponential decay fit. Therefore, a Gaussian function *g*^(1)^(τ) ∼ exp(−τ^2^/) best describes the decay dynamics of the IX_T_ and IX_S_ excited under 400 μW pump power at 90 K. Using this expression for fitting, the dephasing times are found to be *T*_2_ = 0.535 ps for IX_S_ and *T*_2_ = 0.575 ps for IX_T_.

**Figure 3:**
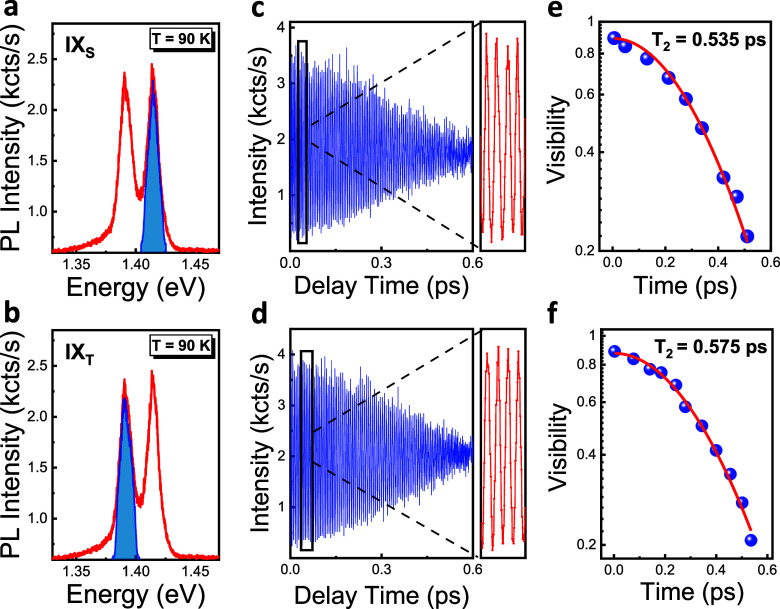
Dephasing time studies of the spin-singlet and spin-triplet IXs using a home-built Michelson interferometer. (a, b) The filtered PL spectra of IX_S_ (a) and IX_T_ (b) with a 532 nm CW laser excitation under 400 μW pump-power at 90 K. Blue regions in both figures show the filtered PL out of the whole spectrum (red line) sent to the Michelson interferometer. (c, d) Corresponding PL interferogram of the first order coherence function *g*^(1)^(τ) of IX_S_ and IX_T_ of a WSe_2_–MoSe_2_ heterobilayer. (e, f) The fringe visibilities as a function of delay time for IX_S_ and IX_T_, which strongly deviates from a monoexponential decay, showing an inhomogeneous decay that corresponds to a Gaussian fit of *g*^(1)^(τ) ∼ exp(−τ^2^/ ).

Finally, we demonstrate for the first time the quantum beats between the coherently coupled IX_T_ and IX_S_ states of a WSe_2_–MoSe_2_ heterobilayer by recording the first-order autocorrelation function *g*^(1)^(τ) with a Michelson interferometer. To study the coherence properties of the coupled states, we simultaneously sent both IX_T_ and IX_S_ PL emissions to the interferometer. Quantum beats can be observed in the time domain due to the excitation of coherently coupled states, denoted by the symbol IX_S-T_ in the following discussion. One can also observe a beat pattern when the optical transitions from two independent (uncoupled) two-level systems with nearly equal transition energies are coupled to an interferometer. In the case of uncoupled systems, oscillations (beats) are created due to the interfering polarizations of two-level systems externally in the detector, and it is known as polarization interference (PI) in the literature. However, in the case of a V-type three-level system (in our case) with two optical transitions between two excited states and a shared ground state, quantum beats are created via quantum mechanical interference due to the coupling of closely separated transitions. If one does not know the nature of their system, it is almost impossible to differentiate between quantum beat and PI by using linear optical techniques. In our case, we know that the two spin states of IXs are mainly due to the splitting of the MoSe_2_ conduction band. As shown in Figure 1b, IX_S_ and IX_T_ take their holes from the same valence band of the WSe_2_ layer and, thus, have one common ground state. In general, nonlinear techniques, including four-wave mixing with time-resolved^20^ or frequency-resolved detection,^41^ can be utilized to make a clear experimental distinction between quantum beats and PI. As schematically described in Figure 4c, as an extension of V type three-level system,^20^ the four-level system (i.e., two excited states (IX_T_ and IX_S_), including a coupled state (IX_S-T_) and one common ground state (G)) can be utilized to explain the coupling between the IX_T_ and IX_S_. The PL intensities of IX_T_ and IX_S_ should be almost identical to maximize the beats’ resolution and the interference’s contrast. Otherwise, as can be seen from the 3.5 K PL spectra in Supporting Information, Figures S2 and S3, the beat resolution will be reduced. To bring those two intensities close to each other, different control knobs can be utilized, such as electrostatic gating,^4,15,18^ temperature,^4,14,16,42^ and pump power of the excitation laser.^15^ As can be seen from our temperature-dependent PL study in Supporting Information, Figure S1, and also from other previous studies, the IX_S_ intensity increases with temperature, whereas the IX_T_ intensity decreases. Therefore, we raised the temperature to 90 K, where the intensities of the IX_T_ and IX_S_ emissions were nearly equal (Figures 4a and S1b). The PL interferogram in Figure 4b exhibits the quantum beat pattern with three visible fringes before it goes out of phase. The dephasing time of the coupled IX_S-T_ state is determined by fitting the beat envelope using the first-order autocorrelation expression *g*^(1)^(τ) = *I*_0_(1 + *A* exp – |τ|/*T*_2_)), resulting in a dephasing time of *T*_2_ = 0.29 ± 0.05 ps. The corresponding homogeneous line width of 9.47 meV is almost equal to the sum of the homogeneous line widths of IX_T_ (4.77 meV) and IX_S_ (5.13 meV) and indicates that spectral fluctuations giving rise to inhomogeneous broadening in each line width are uncorrelated.^43^ The details of the line width calculations are given in the Supporting Information, Note 6. The strong damping of the quantum beats in Figure 4b can be attributed to these uncorrelated fluctuations similar to the one observed for the inhomogeneously broadened exciton and trion spectra of MoSe_2_ in four-wave mixing experiments.^28^ The quantum beat period (*T*_IX_S-T__) can also be obtained from the same interferogram by taking the difference between two fringe maxima or minima, as shown in Figure 4d. The average quantum beat period *T*_IX_S-T__ = 195 ± 17 fs corresponds to an energy separation of Δ*E*_IX_S-T__ = 2πℏ/*T*_IX_S-T__ ∼ 21.3 ± 1.9 meV, which is in close agreement with the Δ*E*_IX_S-T__ value of ∼23.13 meV obtained directly from the time-integrated PL spectrum in Figure 4a and confirms the coherent nature of the coupling between these states. The details of the energy separation calculation by using the measured *T*_IX_S-T__ are given in Supporting Information, Note 7. We also note that the *T*_2_ and *T* values have been measured in various material platforms as well. The *T*_2_ and *T* values on the order of a few ps were measured for the quantum beats of different quasi-particles in quantum wells.^22,23,25^ The coherent coupling between exciton and trion in MoSe_2_ was also studied, and *T*_2_ and *T* values in the range of 250–700 and 132–175 fs were found, respectively.^27−29^ Moreover, *T*_2_ = 100–200 fs and *T* = 116 fs for the coupling of two anisotropic excitons in ReS_2_ have been found in a recent study.^30^

**Figure 4:**
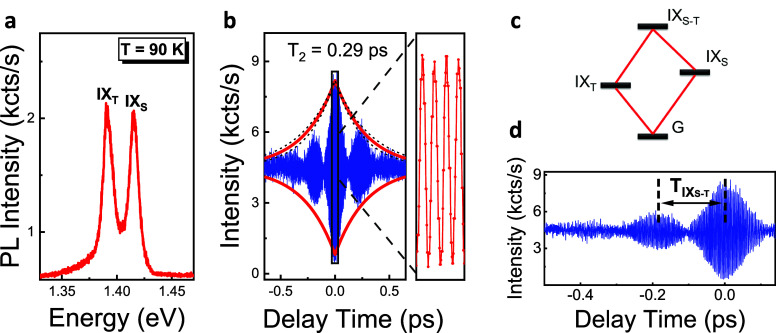
Quantum beat interferometry of spin-singlet and spin-triplet IXs of WSe_2_–MoSe_2_ heterobilayer. (a) PL spectra of the IX_T_ and IX_S_ recorded with 532 nm CW laser excitation under 400 μW pump power at 90 K. (b) Quantum beat interferogram produced by sending both the IX_T_ and IX_S_ emissions simultaneously into the Michelson interferometer. The red-lined beat envelope shows the best fit of the decay that corresponds to *T*_2_ = 0.29 ± 0.05 ps for the spin-singlet and spin-triplet IX coherence. The fit is obtained by using the expression *g*^(1)^(τ) = *I*_0_(1 + *A* exp (−|τ|/*T*_2_)). The dashed lines show the error interval when fitting the beat interferogram via the above expression, which is given by ±0.05 ps. (c) The four-level diamond system showing the coherent coupling between spin-singlet and spin-triplet IX states. (d) The quantum beat period (*T*_IX_S-T__) is obtained from the same interferogram by taking the difference between two fringe maxima.

The fast decay of coherent coupling stems from the dephasing of the IXs itself. To further gain insight into the underlying dephasing mechanisms of the quantum beating between spin-singlet and spin-triplet IXs, we performed both temperature- and excitation pump power-dependent interferometric measurements using two different excitation photon energies (2.33 and 1.7 eV). For temperature-dependent quantum beat measurements, the pump power of the excitation laser was kept fixed at 400 μW for off-resonant excitation (2.33 eV) and 100 μW for near-resonant excitation of 1.7 eV. The resonantly enhanced PL intensities of the two IX species under low pump power of resonant excitation compared to high pump power of off-resonant excitation are mainly due to the maximum absorption in the WSe_2_. The increased amount of intralayer excitons increases the number of excited IXs and, in turn, it would cause the enhanced radiative recombination rate and more intense light emission. Here, two different mechanisms can contribute to the dephasing of IXs: excitation-induced IX–IX scattering and low-energy acoustic phonon interaction. A recent study shows that the main dephasing mechanism for both IX species below 150 K is the low energy acoustic phonon interaction of IX_T_ and IX_S_ with the corresponding Huang–Rhys factors of 1.70 and 1.85, respectively.^10^ Another study also predicts a  temperature dependence for excitation-induced dephasing for IXs.^44^ Considering the results of these two studies together, from our temperature-dependent quantum beat measurements in Figures S2 and S3 and the overview graph in Figure 5a, we can conclude that the fast decay of coherent coupling at 90 K is mainly driven by the strong low-energy acoustic phonon interaction, whereas at 3.5 K, it is driven by the IX-IX interaction. Starting from 20 K, we also note that the IX_T_ PL emission dominates the low-temperature PL spectra when the temperature is further decreased (Figures S2 and S3). Therefore, we could not clearly observe quantum beat patterns at these low temperatures because of the near-zero interference contrast of IX species. Accordingly, we can estimate that the dephasing time of residual coupling between IX species at these low temperatures is dominated or determined by the dephasing time of IX_T_ itself. To gain more insight into the coherent coupling at low temperatures (3.5–20 K) and the nature of the interactions, one can utilize the electrostatic gating^4^ and equate the intensities of these IX species to obtain a reasonable quantum beat pattern.

**Figure 5:**
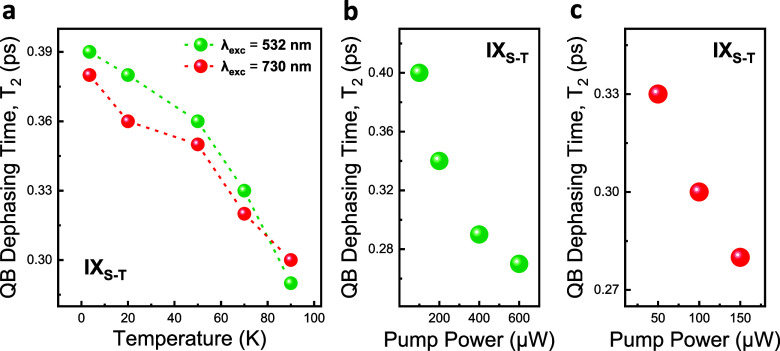
Temperature and pump-power dependence of quantum beat (QB) dephasing. (a) Temperature dependence of the quantum beat dephasing under 532 nm laser excitation with 400 μW pump power (green circles) and 730 nm laser with 100 μW pump power (red circles). (b, c) Pump-power dependence of the quantum beat dephasing under 532 (b) and 730 nm (c) laser excitations. Data are recorded at 90 K in (b) and (c).

To study the effect of excitation laser power on the dephasing times of quantum beats between IX_T_ and IX_S_, we also performed excitation pump power-dependent interferometric measurements at 90 K using two different excitation photon energies. We performed quantum beat measurements first under 532 nm (2.33 eV) CW laser excitation in the pump power range of 100–600 μW. Then, the same quantum beat measurements were also conducted under 730 nm (1.7 eV) laser excitation, which is near-resonant with the intralayer exciton of WSe_2_. Figures S5 and S6 show the PL spectra and corresponding quantum beat interferograms of IX_T_ and IX_S_ at different pump powers under 2.33 and 1.7 eV laser excitations, respectively. The interferograms in Figures S5b and S6b show a pronounced quantum beat pattern with up to five visible fringes. The PL emission intensities of both IX species are resonantly enhanced compared to off-resonant 532 nm excitation, as observed in the previous studies.^6,8,18^ Although we could have performed interferometric measurements under relatively low pump powers (between 50 and 150 μW) compared to off-resonant excitation, we obtained almost the same *T*_2_ values, regardless of the excitation energies whenever the peak intensities of these two PL emissions were comparable (e.g., PL spectra taken under 532 nm excitation with 400 μW and 730 nm excitation with 100 μW in Figure S5 and S6). Our results strongly indicate that a similar number of IXs might have been created in the heterobilayer using two different mechanisms (i.e., strong pumping and resonant enhancement), resulting in similar dephasing times caused by IX-IX scattering. Also, as can be seen from the overview graphs in Figure 5b,c, the dephasing time of the resulting quantum beat signal moderately increases with decreasing pump power. We attribute the fast decay of quantum beats with increasing pump power to the pump power-induced IX-IX scattering.^44^ For the investigated emitter in Figures S5 and S6, however, despite the small dispersion among the extracted beating period (*T*) values under different pump powers, we could not observe a direct correlation between the *T* and excitation pump power for both excitation energies. The excitation intensity independence of the *T* was also observed for the interference of excitons in quantum wells.^45^

In conclusion, we have shown the presence of the coherent coupling between the spin-singlet and spin-triplet interlayer excitons of an hBN encapsulated WSe_2_–MoSe_2_ heterobilayer utilizing quantum beat spectroscopy via a home-built free-space Michelson interferometer. As a clear signature of coherent coupling, the quantum beating signal was observed for the first time between the two spin states of the IX in the time domain. The quantum beat dephasing times up to 400 fs were measured. The calculated energy difference obtained by the measured beating period is in close agreement with the energy separation between the PL emission peaks of spin-singlet and spin-triplet interlayer excitons in the time-integrated PL spectrum and further verifies the coherent nature of the coupling between those states. The observed coherent coupling between two spin states of interlayer excitons can also play an important role in future applications, such as lasing without inversion^46,47^ and electromagnetically induced transparency.^48,49^ Also, quantum beat spectroscopy provides a suitable and convenient technique to investigate the fine structure of IXs not only in our MoSe_2_–WSe_2_ heterobilayer but also for other TMDC heterobilayers, such as WS_2_–WSe_2_^19^. Our results further highlight the important role of coupled quantum states for the future engineering of exciton-based valleytronic and quantum photonic devices.

## Supporting Information Available

Additional data on temperature- and pump power-dependent PL of IX_S_ and IX_T_, temperature- and pump power-dependent quantum beat dephasing time measurements, time-resolved PL spectroscopy of IX_S_ and IX_T_, spectral line width calculation (PDF)
