# Supplementary material for: Quantum Beats between Spin-Singlet and Spin-Triplet Interlayer Exciton Transitions in WSe2–MoSe2 Heterobilayers
Source: Nano Lett. 2024 Apr 19;24(19):5767–73. doi: 10.1021/acs.nanolett.4c00831 (PMC11100286; doi:10.1021/acs.nanolett.4c00831)
Supplement: Supplementary file 1 — nl4c00831_si_001.pdf [file nl4c00831_si_001.pdf]

Quantum beats between spin-singlet and spin-triplet interlayer exciton transitions in WSe_2-$MoSe_2$ heterobilayers

Supporting Information

Mehmet Atıf Durmuş[1], and Ibrahim Sarpkaya[*1]

[1] Bilkent University UNAM – National Nanotechnology Research Center, Ankara 06800, Turkey

[*] Corresponding author. Email: sarpkaya@unam.bilkent.edu.tr

MATERIALS AND METHODS

Sample Preparation

The $MoSe_2$, WSe_2, and hBN layers of the heterostructure were first mechanically exfoliated onto polydimethylsiloxane (PDMS) from their bulk crystal (purchased from 2D Semiconductors). Before fabrication, their thicknesses are evaluated using their color contrast in the optical microscope and then verified by PL-spectroscopy. As it was demonstrated in our previous work[1], the edge identification approach[2] was used to determine the lattice directions of the exfoliated TMD monolayers. After the determination of the zigzag edges, the $MoSe_2$ and WSe_2 monolayers were successfully stacked and aligned ($\theta \sim 60°$) onto a ~100 nm Au-coated Si/SiO_2 substrate using the home-built dry transfer setup.

Low-Temperature Optical Spectroscopy

The low temperature (3.5 K) PL measurements were carried out utilizing the home-built μ-PL setup. Samples were mounted on top of a xyz nanopositioner in the closed-cycle cryostat (Attodry 1000). The picosecond pulsed laser diodes operating at 532 nm and 730 nm under both CW and the pulsed mode were used as an excitation source. The PL emission was collected through a low-temperature compatible microscope objective (0.82 NA) and filtered by a 550 nm long-pass filter (Thorlabs FEL0550). The combination of a 750 mm focal length spectrograph (Princeton Instrument SpectraPro HRS-750) and a liquid nitrogen cooled silicon charge-coupled device (CCD) camera was used to record the PL spectra. A temperature controller (Lake Shore Model 335) was utilized for the temperature-dependent studies. The built-in superconducting magnet

inside the cryostat was used to conduct magneto-PL studies in the range of 0 T to 9 T in Faraday geometry. The dephasing time measurements are done by sending the emission through the home-built Michelson interferometer using a single-mode fiber. According to the interlayer exciton PL characteristics, the PL emission was filtered either by an 850 nm long-pass filter (Thorlabs FELH0850) for the quantum beating interferogram, or 880 nm (Thorlabs FBH880) and 905 nm hard coated (FBH905-10) band-pass filters for individual spin singlet and triplet PL interferograms, respectively.

Quantum Beat Spectroscopy via a Michelson Interferometer

To measure the dephasing time T_2 of individual IX states and observe the quantum beating between these states, a home-built Michelson interferometer with two moveable retro mirrors was constructed. Both the retro mirror on the DC motorized stage (Thorlabs DDS050) with a step size of 5 μm and the retro mirror on the piezo motor stage (Thorlabs PIA13) with a step size of 20 nm could be controlled using a LabVIEW program via appropriate control boxes (Thorlabs KBD101 and TIM101). First-order correlation function $g^{(1)}(\tau)$ measurements were done by using the piezo stage that can resolve the fringes and the data collection was done by using PicoQuant HydraHarp 400 along with a silicon single photon avalanche diode (SPAD) with a photon timing resolution of 50 ps (Micro Photon Devices (MPD)-PDM series). Using the maxima and minima of various fringes, the visibility as a function of delay time was obtained. Due to the inhomogeneous decay from IX_T and IX_S, a Gaussian fit $g^{(1)}(\tau) \sim \exp(-\tau^2/(\frac{T_2}{\sqrt{\pi/2}})^2$ was used to fit the data and their T_2 values were obtained. For the quantum beating interferogram, the decay fit and T_2 values were obtained by using $g^{(1)}(\tau) = I_0(1 + A\exp(-|\tau|/T_2))$.

Supplementary Note 1: Temperature dependence of the PL spectrum of IX spin states

Figure S1a shows the temperature dependence of the IX_T and IX_S PL emission lines. As the temperature increases, the high-energy IX_S state is thermally populated and gets more pronounced at high temperatures. Also, both emission peaks show a redshift with the increased temperature. Similar trends were also observed in the previous studies[3–6]. In **Figure S1b,** we plot the extracted PL intensity ratio of IX_S to IX_T at various temperatures. As can be seen from the figure, this ratio increases with increasing temperature and takes the value of ~1 at 90 K.

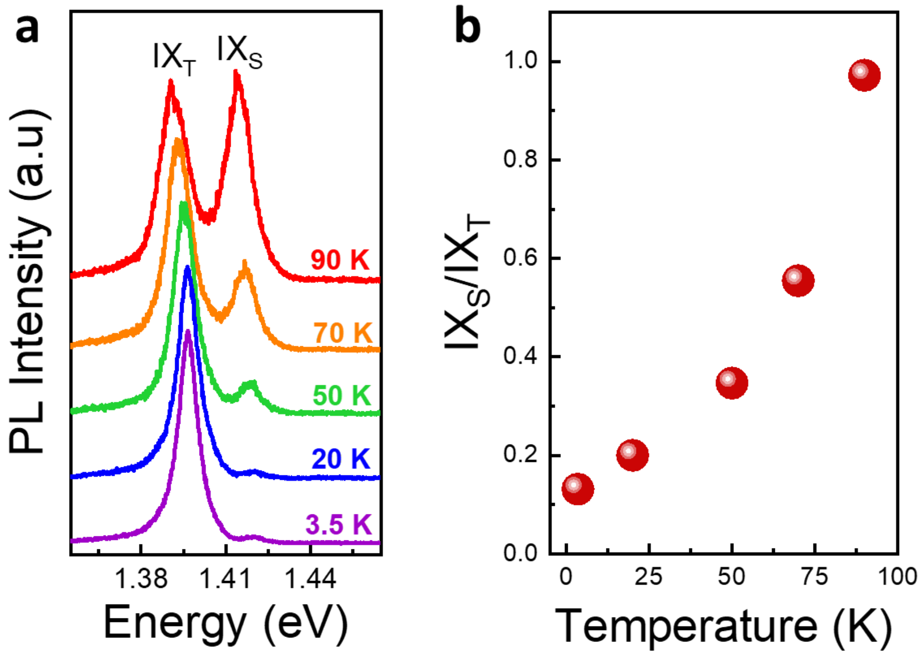

Figure S1: Temperature dependence of spin-singlet and spin-triplet IX PL emission. (a) Temperature-dependent PL spectra of the spin states of IXs. (b) Temperature dependence of the PL intensity ratio of spin-singlet to spin-triplet states of the IXs. Data are recorded under a 532 nm CW laser with 400 μW pump power.

Supplementary Note 2: Temperature-dependent quantum beat interferometry

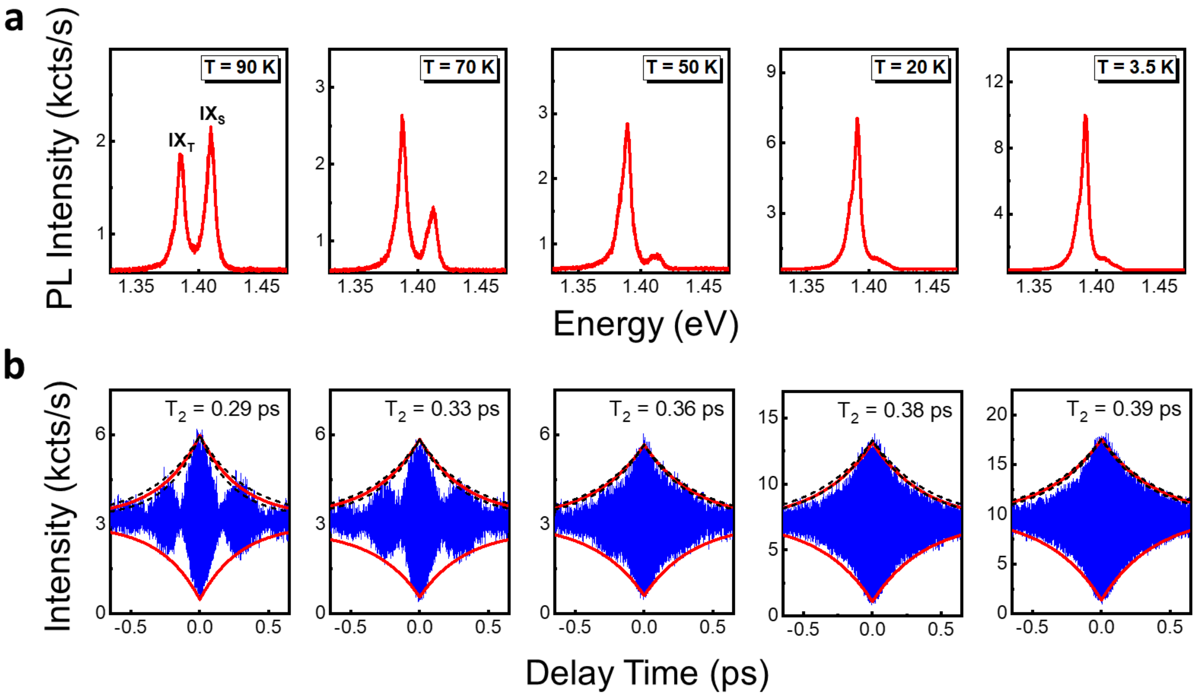

Figure S2: Temperature dependence of the quantum beat interferometry of spin-singlet and spin-triplet IXs of WSe_2-$MoSe_2$ heterobilayers under 532 nm laser excitation. (a) Temperature-dependent PL spectra of the IX_T and IX_S recorded under 400 μW pump power. (b) Temperature-dependent quantum beating interferograms of the IX_T and IX_S, produced by sending both emissions simultaneously into the Michelson interferometer. The red-lined beat envelope shows the best fit of the decay that corresponds to the dephasing time for the spin-singlet and spin-triplet IX coherence. The fit is obtained by using the expression $g^{(1)}(\tau) = I_0(1 + A\exp(-|\tau|/T_2))$. The dashed lines show the error interval when fitting the beat interferogram via the above expression, which is given by ± 0.05 ps, ± 0.04 ps, ± 0.03 ps, ± 0.03 ps, and ± 0.03 ps, from 90 K to 3.5 K, respectively.

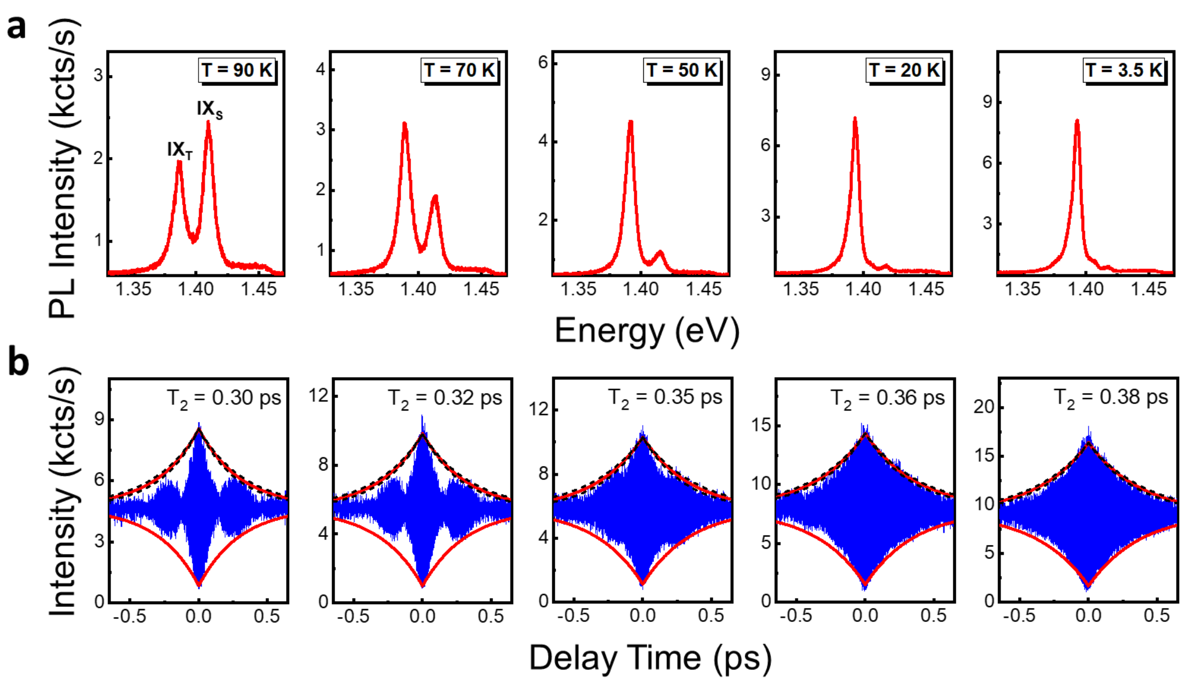

Figure S3: Temperature dependence of the quantum beat interferometry of spin-singlet and spin-triplet IXs of WSe_2-$MoSe_2$ heterobilayers under 730 nm laser excitation. (a) Temperature-dependent PL spectra of the IX_T and IX_S recorded under 100 μW pump power. (b) Temperature-dependent quantum beating interferograms of the IX_T and IX_S, produced by sending both emissions simultaneously into the Michelson interferometer. The red-lined beat envelope shows the best fit of the decay that corresponds to the dephasing time for the spin-singlet and spin-triplet IX coherence. The fit is obtained by using the expression $g^{(1)}(\tau) = I_0(1 + A\exp(-|\tau|/T_2))$. The dashed lines show the error interval when fitting the beat interferogram via the above expression, which is given by ± 0.03 ps, ± 0.03 ps, ± 0.04 ps, ± 0.03 ps, and ± 0.03 ps, from 90 K to 3.5 K, respectively.

Supplementary Note 3: Pump-power dependence of the PL spectrum of IX spin states

Figures S4a and **S4b** show the full evolution of the PL spectrum under various excitation pump powers at 3.5 K and 90 K, respectively. At low temperatures (3.5 K) and under low excitation power, the sharp and narrow PL emission lines of a few moiré localized IXs dominate the PL spectrum. When the excitation pump power was increased, the narrow and sharp emission lines evolved into a broad ensemble band of low-energy configuration of interlayer excitons (IX_T). Once the pump power was further increased, the high energy spin-split state of IX (IX_S) also started to fill out due to the phase space-filling effect. The PL intensity of IX_S continues to increase with increased pump power, whereas IX_T intensity saturates[7]. The PL emission signature related to the IX_S state appeared on the high energy side of the PL spectrum with an energy separation of ~24 meV from IX_T. Also, the PL emission lines of both IX species show a blueshift with increasing pump power as a signature of repulsive dipolar interaction[8,9] between interlayer excitons.

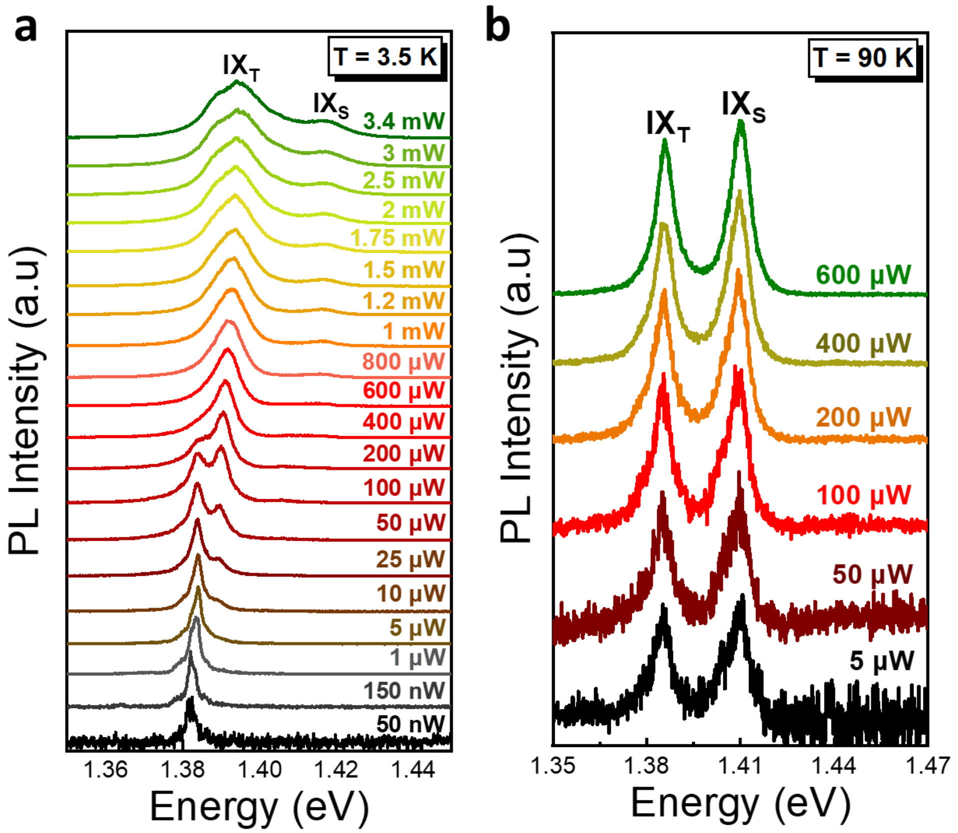

Figure S4: Pump power-dependent PL of the IX species of a WSe_2-$MoSe_2$ heterobilayer. Pump power-dependent PL at 3.5 K (a) and 90 K (b) under 532 nm laser excitation.

Supplementary Note 4: Power-dependent quantum beat interferometry

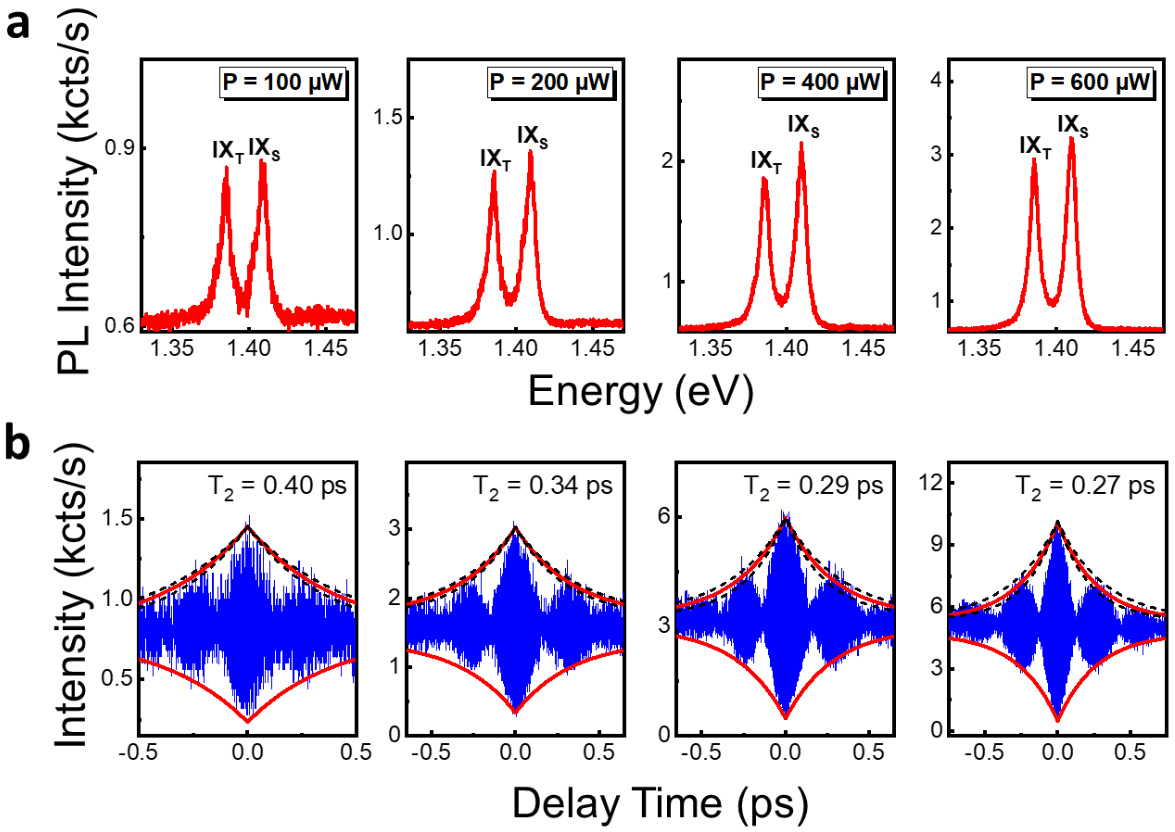

Figure S5: Pump-power dependence of the quantum beat interferometry of spin-singlet and spin-triplet IXs of WSe_2-$MoSe_2$ heterobilayers under 532 nm laser excitation. (a) Power-dependent PL spectra of the IX_T and IX_S recorded at 90 K. (b) Power-dependent quantum beating interferograms of the IX_T and IX_S, produced by sending both emissions simultaneously into the Michelson interferometer. The red-lined beat envelope shows the best fit of the decay that corresponds to the dephasing time for the spin-singlet and spin-triplet IX coherence. The fit is obtained by using the expression $g^{(1)}(\tau) = I_0(1 + A \exp(-|\tau|/T_2))$. The dashed lines show the error interval when fitting the beat interferogram via the above expression, which is given by ± 0.05 ps, ± 0.04 ps, ± 0.05 ps, and ± 0.05 ps from 100 μW to 600 μW, respectively.

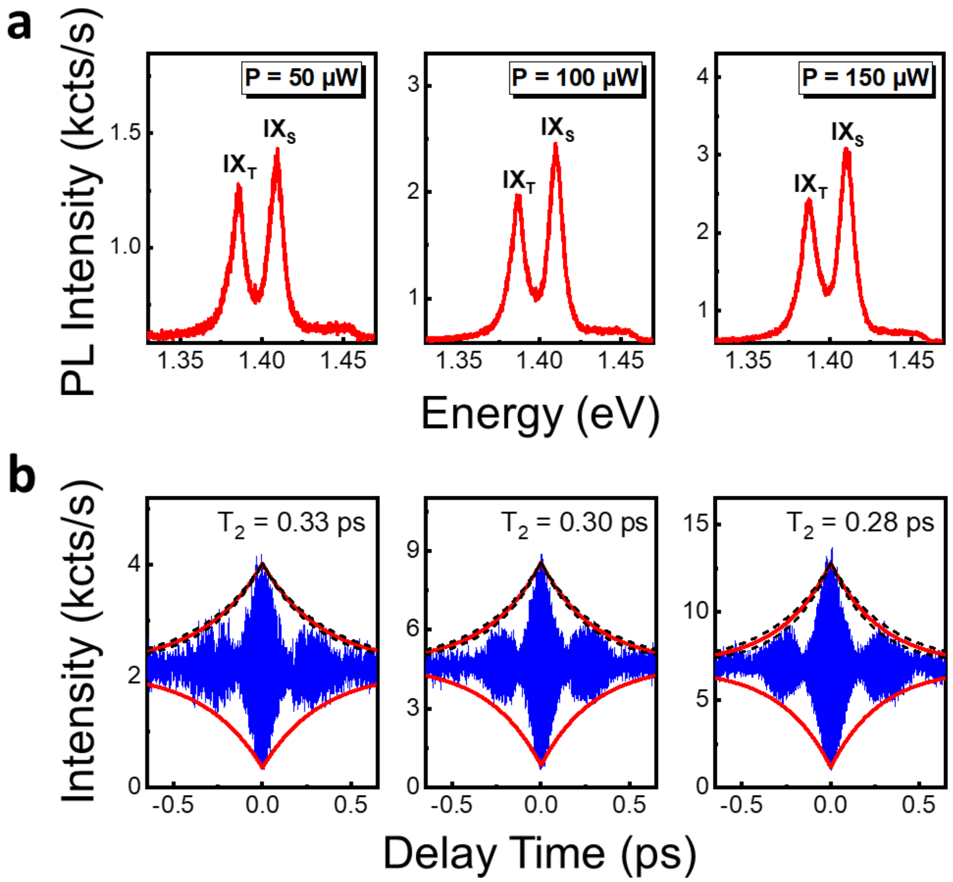

Figure S6: Pump-power dependence of the quantum beat interferometry of spin-singlet and spin-triplet IXs of WSe_2-$MoSe_2$ heterobilayers under 730 nm laser excitation. (a) Power-dependent PL spectra of the IX_T and IX_S recorded at 90 K. (b) Power-dependent quantum beating interferograms of the IX_T and IX_S, produced by sending both emissions simultaneously into the Michelson interferometer. The red-lined beat envelope shows the best fit of the decay that corresponds to the dephasing time for the spin-singlet and spin-triplet IX coherence. The fit is obtained by using the expression $g^{(1)}(\tau) = I_0(1 + A\exp(-|\tau|/T_2))$. The dashed lines show the error interval when fitting the beat interferogram via the above expression, which is given by ± 0.03 ps, ± 0.03 ps, and ± 0.04 ps from 50 μW to 150 μW, respectively.

Supplementary Note 5: Time-resolved PL spectroscopy of IX_T and IX_S

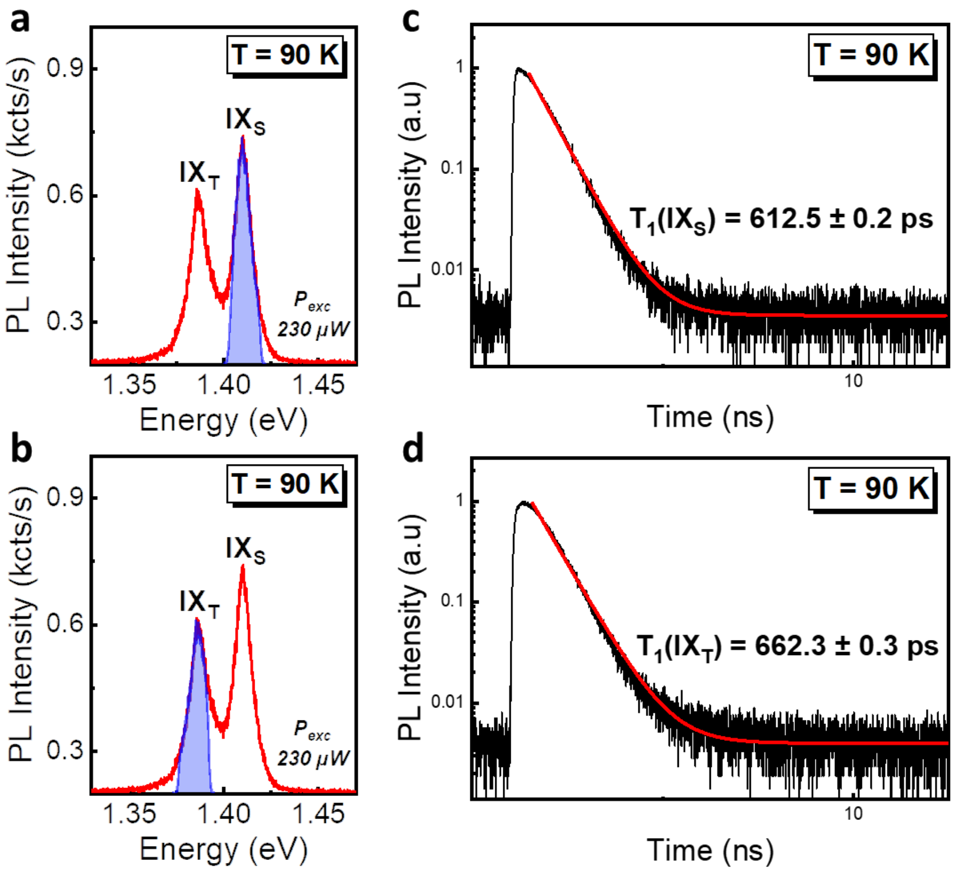

Figure S7: Time-resolved PL spectroscopy of spin-singlet and spin-triplet interlayer excitons of WSe_2-$MoSe_2$ heterobilayers. (a-b) PL spectra of IX_T and IX_S at 90 K under 80-MHz pulsed laser excitation at 532 nm with an incident power of P = 230 μW. Blue areas show the filtered parts of the PL spectra. Hard-coated band-pass filters with wavelengths of 880 nm and 905 nm are used for IX_S and IX_T, respectively. (c-d) The time-resolved PL intensity of filtered IX_S and IX_T emissions as a function of delay time after pulsed excitation at 532 nm. A monoexponential decay of the lifetime is indicated by the red-lined fit, yielding $T_1 = 612.5 \pm 0.2$ ps for IX_S and $T_1 = 662.3 \pm 0.3$ ps for IX_T. The same experiment has also been performed with P = 50 μW excitation power, which yielded $T_1 = 660.0 \pm 2.0$ ps for IX_S and $T_1 = 710.0 \pm 0.5$ ps for IX_T.

Supplementary Note 6: Linewidth calculation using the dephasing time values

The resulting visibility semilog plots from the interferograms of the IX_S and IX_T (**Figure 3** in the main text) show a Gaussian type of decay in the fringe visibilities. A Gaussian-type emission's spectral line width (Γ) can be calculated using the well-known expression $\Gamma = \frac{\hbar\sqrt{8\pi ln2}}{T_2}$. The dephasing time ($T_2$) here can be expressed with the relation $\frac{1}{T_2} = \frac{1}{2T_1} + \frac{1}{T_2^*}$, where T_2^* is the pure dephasing time and T_1 is the radiative lifetime of the IXs. As can be seen from **Figure S7**, the radiative lifetimes of IX_T and IX_S at 90 K (~600 – 700 ps) are almost three orders of magnitude larger than their dephasing times (~300 – 400 fs), meaning that contributions from the radiative lifetimes to the spectral line width are negligible. Therefore, from the above relation, approximation of $T_2^* \sim T_2$ can be made and used in the spectral line width calculation. The calculated linewidth of coupled state is given by:

$$\Gamma_{IX_{S-T}} \sim \frac{\hbar\sqrt{8\pi ln2}}{T_2(IX_{S-T})} = \frac{(6.582 \times 10^{-16}\ eV.s)\sqrt{8\pi ln2}}{0.29 \times 10^{-12}\ s} = 9.47\ meV$$

The line widths of the IX_S and IX_T can be calculated from their experimentally measured individual dephasing times. As indicated in the main text, their dephasing times are given by 0.535 ps and 0.575 ps, respectively. Using them in the above line width equation, their homogenous line widths can be calculated as follows.

$$\Gamma_{IX_S} \sim \frac{\hbar\sqrt{8\pi ln2}}{T_2(IX_S)} = \frac{(6.582 \times 10^{-16}\ eV.s)\sqrt{8\pi ln2}}{0.535 \times 10^{-12}\ s} = 5.13\ \text{meV}$$

$$\Gamma_{IX_T} \sim \frac{\hbar\sqrt{8\pi ln2}}{T_2(IX_T)} = \frac{(6.582 \times 10^{-16}\ eV.s)\sqrt{8\pi ln2}}{0.575 \times 10^{-12}\ s} = 4.77\ \text{meV}$$

$$\Gamma_{IX_{S-T}} \sim \Gamma_{IX_S} + \Gamma_{IX_T} \rightarrow 9.47\ \text{meV} \sim 9.89\ \text{meV}$$

The calculated homogeneous linewidth of coupled state 9.47 meV is almost equal to the sum of the homogeneous linewidths of IX_T (4.77 meV) and IX_S (5.13 meV) and indicates that spectral fluctuations giving rise to inhomogeneous broadening in each line widths are uncorrelated[10]. The strong damping of the quantum beats in **Figure 4b** of the main text can be attributed to these uncorrelated fluctuations similar to the one observed for the inhomogenously broadened exciton and trion spectra of $MoSe_2$ in four-wave mixing experiments[11]. **Figure S8** displays the PL spectral

line widths of the IX_S (9.97 meV) and IX_T (11.64 meV) obtained from the time-integrated PL spectrum. It clearly shows that inhomogeneous broadening contributes to the PL line widths and can be attributed to the charge fluctuations in the vicinity of the IXs.

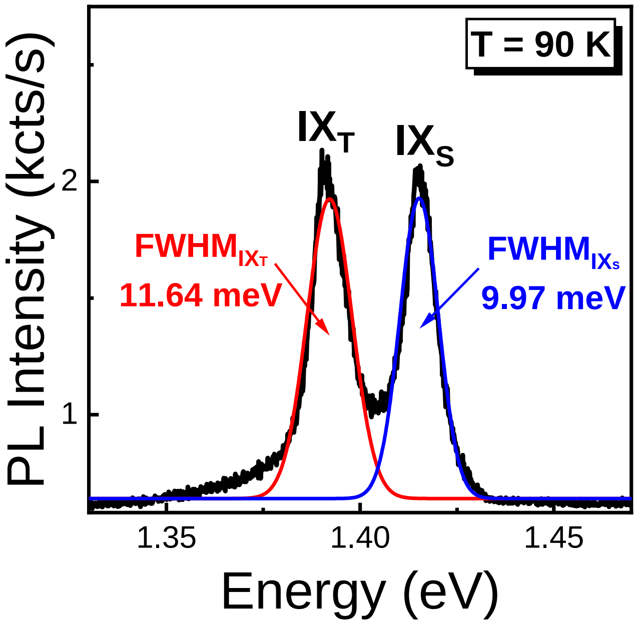

Figure S8: PL spectrum and spectral line widths of spin-singlet and spin-triplet IXs at 90 K. Red and blue-lined Gaussian fits show the individual peak fits of IX_T and IX_S, respectively. Data are recorded under 532 nm CW laser excitation with an incident power of 400 μW.

Supplementary Note 7: Calculation of the energy splitting between the IX_T and IX_S via measured beat period

The energy splitting between IX_T and IX_S states can be calculated using the expression $\Delta E_{IX_{S-T}} = 2\pi\hbar/T_{IX_{S-T}}$. Here, $T_{IX_{S-T}}$ is the average quantum beat period and can be directly determined from the difference between the fringe maxima or minima in the quantum beat interferogram. We determined the average beat period as $T_{IX_{S-T}} = 195 \pm 17$ fs, which can be used to calculate the energy splitting as follows.

$$\Delta E_{IX_{S-T}} = \frac{2\pi\hbar}{T_{IX_{S-T}}} = \frac{2\pi(6.582 \times 10^{-16}\ eV.s)}{195 \pm 17 \times 10^{-15}\ s} = 21.3 \pm 1.9 \text{ meV}$$

The calculated energy difference and the energy difference obtained directly from the time integrated PL spectrum can be compared to further confirm the nature of the coupling between the IX_S and IX_T. **Figure S9** shows the PL spectrum of the both spin states of the IX at 90 K. The separation between the centers of the IX_T and IX_S PL peaks is given by $\Delta E_{IX_{S-T}} \sim 23.13$ meV. It is evident that the calculated and measured energy separation values are in close agreement, confirming the coherent nature of the coupling between these IX states.

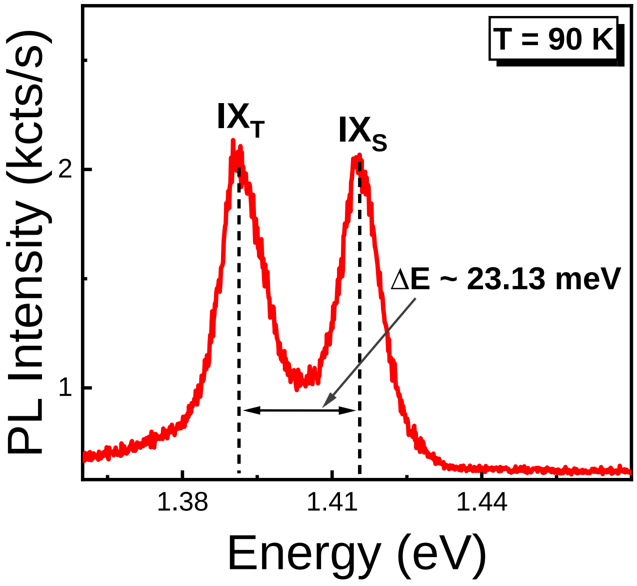

Figure S9: PL spectrum of the spin states of IX and the experimental energy separation between them at 90 K. Data are recorded under 532 nm CW laser excitation with an incident power of 400 μW.

REFERENCES

(1) Durmuş, M. A.; Demiralay, K.; Khan, M. M.; Atalay, Ş. E.; Sarpkaya, I. Prolonged Dephasing Time of Ensemble of Moiré-Trapped Interlayer Excitons in WSe2-MoSe2 Heterobilayers. *Npj 2D Mater. Appl.* **2023**, *7* (1), 1–8.

(2) Guo, Y.; Liu, C.; Yin, Q.; Wei, C.; Lin, S.; Hoffman, T. B.; Zhao, Y.; Edgar, J. H.; Chen, Q.; Lau, S. P.; Dai, J.; Yao, H.; Wong, H.-S. P.; Chai, Y. Distinctive In-Plane Cleavage Behaviors of Two-Dimensional Layered Materials. *ACS Nano* **2016**, *10* (9), 8980–8988.

(3) Ciarrocchi, A.; Unuchek, D.; Avsar, A.; Watanabe, K.; Taniguchi, T.; Kis, A. Polarization Switching and Electrical Control of Interlayer Excitons in Two-Dimensional van Der Waals Heterostructures. *Nat. Photonics* **2019**, *13* (2), 131–136.

(4) Zhang, L.; Gogna, R.; Burg, G. W.; Horng, J.; Paik, E.; Chou, Y.-H.; Kim, K.; Tutuc, E.; Deng, H. Highly Valley-Polarized Singlet and Triplet Interlayer Excitons in van Der Waals Heterostructure. *Phys. Rev. B* **2019**, *100* (4), 041402.

(5) Liu, E.; Barré, E.; van Baren, J.; Wilson, M.; Taniguchi, T.; Watanabe, K.; Cui, Y.-T.; Gabor, N. M.; Heinz, T. F.; Chang, Y.-C.; Lui, C. H. Signatures of Moiré Trions in WSe2/MoSe2 Heterobilayers. *Nature* **2021**, *594* (7861), 46–50.

(6) Hanbicki, A. T.; Chuang, H.-J.; Rosenberger, M. R.; Hellberg, C. S.; Sivaram, S. V.; McCreary, K. M.; Mazin, I. I.; Jonker, B. T. Double Indirect Interlayer Exciton in a MoSe2/WSe2 van Der Waals Heterostructure. *ACS Nano* **2018**, *12* (5), 4719–4726.

(7) Joe, A. Y.; Jauregui, L. A.; Pistunova, K.; Mier Valdivia, A. M.; Lu, Z.; Wild, D. S.; Scuri, G.; De Greve, K.; Gelly, R. J.; Zhou, Y.; Sung, J.; Sushko, A.; Taniguchi, T.; Watanabe, K.; Smirnov, D.; Lukin, M. D.; Park, H.; Kim, P. Electrically Controlled Emission from Singlet and Triplet Exciton Species in Atomically Thin Light-Emitting Diodes. *Phys. Rev. B* **2021**, *103* (16), L161411.

(8) Li, W.; Lu, X.; Dubey, S.; Devenica, L.; Srivastava, A. Dipolar Interactions between Localized Interlayer Excitons in van Der Waals Heterostructures. *Nat. Mater.* **2020**, *19* (6), 624–629.

(9) Nagler, P.; Plechinger, G.; Ballottin, M. V.; Mitioglu, A.; Meier, S.; Paradiso, N.; Strunk, C.; Chernikov, A.; Christianen, P. C. M.; Schüller, C.; Korn, T. Interlayer Exciton Dynamics in a Dichalcogenide Monolayer Heterostructure. *2D Mater.* **2017**, *4* (2), 025112.

(10) Cundiff, S. T. Effects of Correlation between Inhomogeneously Broadened Transitions on Quantum Beats in Transient Four-Wave Mixing. *Phys. Rev. A* **1994**, *49* (4), 3114–3118.

(11) Hao, K.; Xu, L.; Nagler, P.; Singh, A.; Tran, K.; Dass, C. K.; Schüller, C.; Korn, T.; Li, X.; Moody, G. Coherent and Incoherent Coupling Dynamics between Neutral and Charged Excitons in Monolayer MoSe2. *Nano Lett.* **2016**, *16* (8), 5109–5113.
